# Parents’ Perceptions of Children’s and Adolescents’ Use of Electronic Devices to Promote Physical Activity: Systematic Review of Qualitative Evidence

**DOI:** 10.2196/44753

**Published:** 2023-07-20

**Authors:** María Eugenia Visier-Alfonso, Mairena Sánchez-López, Beatriz Rodríguez-Martín, Abel Ruiz-Hermosa, Raquel Bartolomé-Gutiérrez, Irene Sequí-Domínguez, Vicente Martínez-Vizcaíno

**Affiliations:** 1 Faculty of Nursing University of Castilla-La Mancha Cuenca Spain; 2 School of Education University of Castilla-La Mancha Ciudad Real Spain; 3 Faculty of Occupational Therapy, Logopedia and Nursing University of Castilla-La Mancha Toledo Talavera de la Reina Spain; 4 Department of Didactics of Musical, Plastic and Body Expression Faculty of Sports and Sciences University of Extremadura Cáceres Spain; 5 Department of Psychology University of Castilla-La Mancha Albacete Spain; 6 Health and Social Research Center University of Castilla-La Mancha Cuenca Spain

**Keywords:** physical activity, electronic devices, eHealth, parents’ perceptions, children, adolescents, systematic review, qualitative

## Abstract

**Background:**

The use of physical activity (PA) electronic devices offers a unique opportunity to engage children and adolescents in PA. For this age group (2-17 years), parents play a key role in promoting healthy lifestyles and regulating the use of electronic devices. Therefore, parents’ perceptions of the use of electronic devices for PA in children and adolescents are critical for efficient intervention.

**Objective:**

The aim of this qualitative systematic review was to improve the understanding of parents’ perceptions of the use of electronic devices for PA in children and adolescents.

**Methods:**

A systematic search of electronic databases (Medline/PubMed, SPORTDiscus, Web of Science, Scopus, OpenGrey, and Deep Blue) was conducted. Studies from inception (2010) to May 2022 were identified. Qualitative studies on the perceptions of healthy children’s and adolescents’ (aged 2-17 years) parents regarding PA interventions performed on electronic devices were included according to the Cochrane Qualitative and Implementation Methods Group Guidance Series and the Enhancing Transparency in Reporting the Synthesis of Qualitative Research (ENTREQ) statement. The Joanna Briggs Institute Qualitative Assessment and Review Instrument was used for methodological validity.

**Results:**

In total, 18 studies with 410 parents, mostly mothers, were included. Parents’ perceptions were grouped into 4 categories: usefulness, advantages, general perceptions (electronic devices for health promotion, preferences for real-life PA, and concerns), and acceptability (barriers and facilitators) of electronic devices for PA. Parents perceived electronic devices as useful for increasing PA, learning new skills, and increasing motivation for PA and valued those devices that promoted socialization and family and peer bonding. In terms of general perceptions, parents had positive attitudes toward PA electronic devices; however, they preferred outdoor and real-life PA, especially for preschoolers and children. Concerns, such as physical and psychological harm, addiction, conflicts, and compliance difficulties, were found. Facilitators were identified as ease of use, appropriate feedback, promotion of socialization, and motivational strategies, such as rewards, challenges, and attractiveness. Barriers, such as discomfort, price, and difficulties in using or understanding electronic devices, were also identified. For older children and adolescents, parents were more concerned about high levels of screen time and setting limits on electronic devices and therefore preferred PA electronic devices rather than traditional ones.

**Conclusions:**

Overall, the participants had positive attitudes toward electronic devices for PA and perceived them as an effective way to promote PA in children and adolescents. They also perceived several benefits of using electronic devices, such as health promotion, increased awareness and motivation, and socialization, as well as barriers, facilitators, and age differences. The results of this study could provide researchers with insights into designing more effective, age-appropriate PA electronic devices for children and adolescents and improving adherence to their use.

**Trial Registration:**

PROSPERO CRD42021292340; https://www.crd.york.ac.uk/prospero/display_record.php?RecordID=292340

## Introduction

Currently, smartphones, tablets, computers, and apps that run on electronic devices have become part of the everyday life of children and adolescents [1]. Most parents allow their children to use their smartphones to play games or watch videos, and almost all children start handling electronic devices before the age of 1 year [2]. In addition, 73% of parents with children aged 9-11 years say that their children use a computer, 68% say that they use gaming devices, 67% say that they use a smartphone, and 78% say that they use a tablet [1]. There are substantial age differences in the use of electronic devices, and usage increases with age, being higher in adolescents, with most of them reporting using electronic devices daily or almost all the time [3]. Traditionally, research on the use of electronic devices has focused on its association with sleep problems, sedentarism, and overweight/obesity [4]. However, with the growth in technology, the use of eHealth (ie, electronic devices with health-related purposes [5], including physical activity [PA] and fitness apps), has increased [6].

Some advantages of using electronic devices to implement PA interventions are that these programs are more flexible, can be tailored to individual needs, and can be delivered anywhere at any time compared to traditional PA interventions [7]. Moreover, electronic devices might make PA more attractive to children and adolescents [8], as well as having other advantages, such as low cost, empowerment of participants, exposure to new information, increased opportunities for social contact, and new opportunities to access health promotion programs [9]. The potential role of apps in improving PA across children and adolescents has been suggested [10], but evidence of the efficacy of PA apps for this age group is still scarce [10,11]. Thus, more research on electronic devices to promote PA in children and adolescents is needed.

Furthermore, early habits track from childhood through adolescence to adulthood [12], making early childhood a crucial period for the acquisition of habits, such as PA. In addition, parents’ behaviors related to PA have been shown to be associated with their children’s health behaviors [13]. Previous research indicates that PA programs that include families are more effective in increasing PA in children [14,15]. Moreover, a meta-analysis by Hammersley et al [16] suggested that eHealth interventions might be more successful when parents are involved as agents of change. Not only health-related behaviors but also screen time and electronic device access and use depend on the individual’s family [17]. Additionally, parents’ attitudes toward electronic devices are associated with different regulation practices, depending on age and the time spent using electronic devices from childhood through adolescence [18]. All these results recommend parents’ involvement in eHealth interventions [19], with the family being a key intervention target [20]. Finally, from a qualitative perspective, Burrows et al [21] found that most parents are interested in an online eHealth family program and that they feel that important features of the program should be easy to use, engaging, and endorsed by a reputable source and should involve their children directly.

To examine the feasibility of PA interventions delivered through electronic devices, before implementing the interventions, it is critical to understand parents’ perceptions of the interventions because parents’ engagement in these activities is a key factor for their success in children [21] and in the regulation and mediation practices that control electronic device use in adolescents [22]. However, to date, no reviews have focused on parents’ opinions and perceptions of eHealth to promote PA in children and adolescents, although this knowledge might be relevant for the design of both PA electronic devices and effective interventions. The aim of this systematic review of qualitative evidence is to increase the understanding of parents’ perceptions of electronic device–based PA interventions in children and adolescents.

## Methods

### Overview

This review was conducted according to the Cochrane Qualitative and Implementation Methods Group Guidance Series [23] and the Enhancing Transparency in Reporting the Synthesis of Qualitative Research (ENTREQ) statement [24]. The review protocol was registered in PROSPERO (CRD42021292340).

### Eligibility Criteria

Studies were eligible for inclusion if they reported qualitative research analyses of the use of electronic devices for PA in healthy children and adolescents. In this study, electronic devices were defined as tools that can receive, store, process, or send digital information, including computers, tablets, smartphones, smart or electronic watches, and virtual reality devices [25]. Studies using qualitative designs with any of the following data collection procedures were eligible for inclusion: interviews, focus groups, or other qualitative data collection procedures, such as observation. Mixed methods studies were included when quantitative and qualitative data were separately reported; however, only data on qualitative analyses were considered. There are different types of electronic devices (ie, activity trackers, video games, smartphone apps) for direct use by children, for use by parents to enhance their children’s PA, or for use by both together.

Studies were excluded if (1) parents were not directly asked; (2) PA interventions referred participants to rehabilitation programs or facilities; (3) populations had developmental disabilities, developmental delays, or cognitive impairment; (4) the electronic device was not designed for use by children or adolescents or for interactive use by parents and children (eg, electronic devices for parents’ use only); and (5) the study was a protocol, review, or meta-synthesis.

### Search Strategy

Two authors (MVA and ARH) independently identified qualitative studies published from the beginning (in 2010) up to May 2022, reporting parents’ perceptions of PA electronic devices. The research objective was addressed with the question framework PerSPecTIF proposed by Booth et al [26]. Both authors systematically searched Medline/PubMed, SPORTDiscus, Web of Science, and Scopus using a search strategy that combined 5 different concepts: “electronic devices,” “physical activity,” “parents,” “qualitative research,” and “children and adolescents.” The free-text terms and Medical Subject Headings (MeSH) terms used to search were restricted to titles/abstracts. Searches for gray literature (eg, unpublished studies) were conducted using OpenGrey and Deep Blue. In addition, the 2 authors screened the reference lists of the papers included. The complete search strategy is presented in Multimedia Appendix 1.

### Study Selection

Search terms were entered into each database, and duplicates were removed. The titles and abstracts retrieved were independently assessed for eligibility for inclusion in the review by 2 authors (MVA and ARH) and coded as “yes,” “no,” or “maybe.” The 2 authors were trained regarding study inclusion/exclusion criteria before completing the coding of abstracts. Any disagreements between the 2 authors were resolved through discussion, and if disagreement persisted, a third author (MSL) was consulted.

### Assessment of Methodological Quality

Papers selected for inclusion were assessed by 2 authors (MVA and MSL) using the 10-item checklist of the Johanna Briggs Institute Qualitative Assessment and Review Instrument (JBI-QARI) [27] for methodological validity prior to inclusion in the review. All items in the checklist were ranked as “yes,” “no,” or “unclear.” Finally, each study was rated overall as “included,” “excluded,” or “seeking further info” [27]. Studies meeting more than 7 items were rated as “included,” studies with items rated as “no” or “unclear” were rated as “seeking further info” and protocols, and corresponding authors were consulted. Studies meeting less than 5 items were rated as “excluded” and removed from the study. Any disagreements between the 2 authors were resolved through discussion, and a third author (BRM) was consulted if disagreement persisted.

### Data Abstraction

Qualitative data were extracted by 2 independent authors (MVA and MSL). Both authors read the papers and extracted key themes and concepts. These were compared, and any differences were resolved through discussion. The following data were extracted from all eligible papers: authors and context, year of publication, location, paradigmatic approach, method of data collection and analysis, data analysis software, participants’ background, sample size and age, recruitment location and method, study aims, intervention or exposure, and main results.

### Data Analysis and Synthesis

First, 2 authors (MVA and MSL) read the papers, extracted key themes and proofs (transcriptions of parents’ verbalizations), and generated categories. A third author (BRM) was consulted if discrepancies arose. Differences were solved through discussion until agreement was reached. To identify common themes and analyze meanings, the meta-aggregation approach [28] was used. This process identifies meanings and common themes in qualitative studies using different methodologies and further extracts those meanings into categories that are then synthesized [29]. Next, MVA synthesized the key themes, meanings, and proofs (transcriptions of parents’ verbalizations) into tables.

## Results

### Study Selection and Characteristics

**Figure 1:**
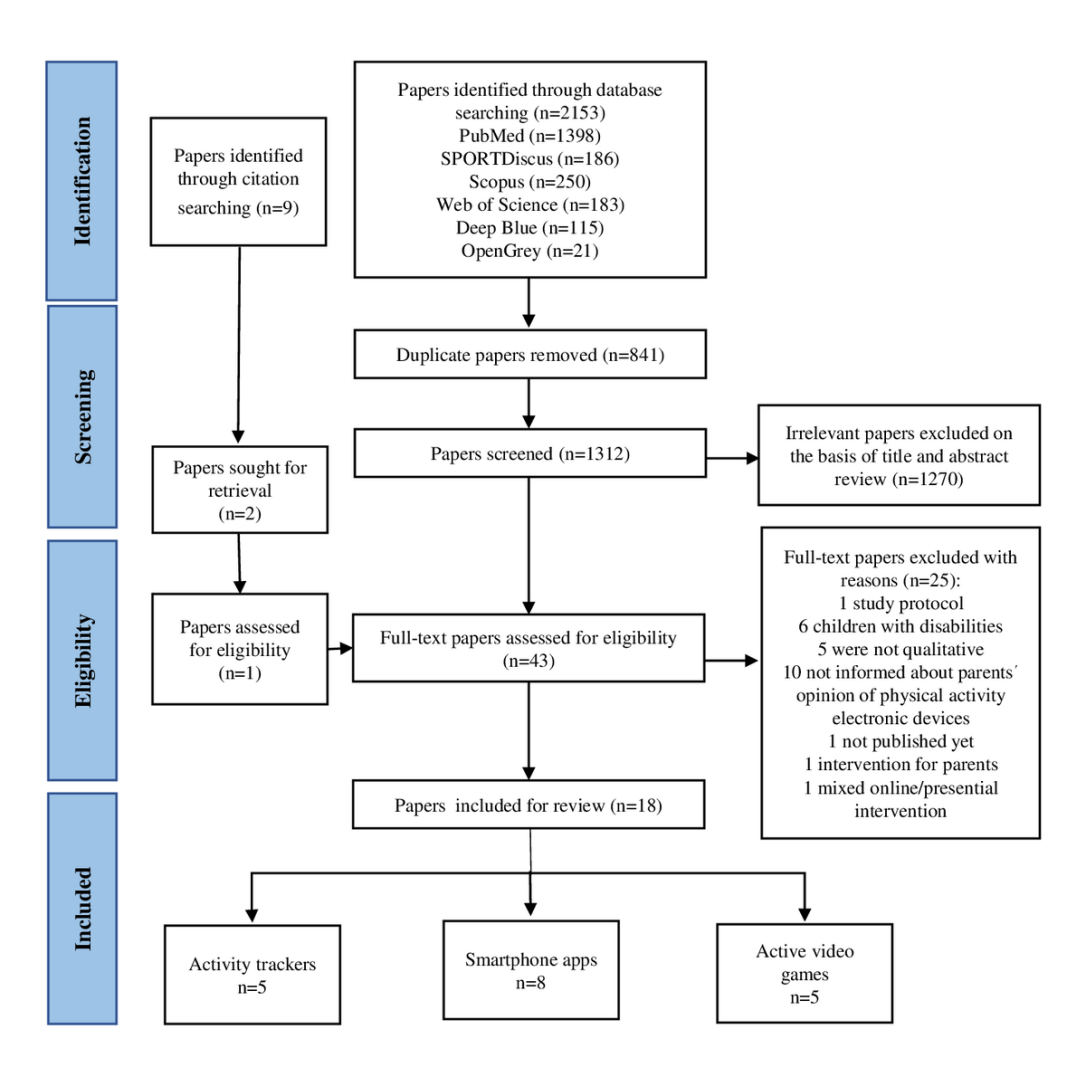
Flowchart of search and selection process.

The electronic search retrieved 2153 records. After the removal of duplicate studies, 1312 (60.9%) papers were reviewed based on the title and abstract. Following this, the full texts of 43 (3.3%) studies were reviewed; 1 (0.1%) additional study was identified after screening the reference lists of eligible papers. Finally, 18 (41%) eligible papers were included using the selection process shown in Figure 1.

The 18 studies selected were published between 2010 and May 2022 and included 410 parents, mostly mothers, of 2-17-year-old children and adolescents (Tables 1-3). Of the 18 studies, 5 (28%) analyzed preschool children, 7 (39%) analyzed school children, 3 (17%) analyzed adolescents, and 3 (17%) did not provide separate results for children and adolescents. For data collection, 12 (67%) studies [30-41] used focus groups with semistructured interviews, 7 (39%) [20,30,42-46] used individual interviews, and 1 (6%) [41] used nonparticipant observation. Regarding the electronic devices analyzed, 5 (28%) studies [20,30,38,41,42] used smartphone apps, 2 (11%) [37,40] used the *Pokémon GO* mobile game, 1 (6%) [45] used mobile text messages, 5 (28%) [31-33,39,44] used activity trackers, 4 (22%) [34-36,43] used active video games, and 1 (6%) [46] used virtual reality.

**Table 1:** Characteristics of included studies (preschoolers).

Author, country	Method of data collection	Method of analysis (software); paradigmatic approach	Participants’ details (background, age, parents’ details)	Place and methods of recruitment
McCloskey et al [20], United States	Individual semistructured telephonic and face-to-face interviews	Thematic analysis, inductive approach (NVivo v.11, QSR International); N/I^a^	Background: low-income families in rural areas Age=3-5 years Parents (telephonic interviews): n=29, mean age N/I, 93% (27/29) mothers Parents (face-to-face interviews): n=31, mean age N/I, 77% (24/31) mothers	Purposive sampling (preschool centers, letters)
Alexandrou et al [30], Sweden	Focus groups, individual interviews	Thematic analysis, inductive approach; N/I	Background: socioeconomically diverse district Age=2.5-3 years Somali parents: n=5, mean age 34 (SD 6.6) years; 100% (5/5) mothers Arabic parents: n=4, mean age 31.2 (SD 2) years, 100% (4/4) mothers Swedish parents: n=6, mean age 35.8 (SD 4.7) years, 83% (5/6) mothers	Purposive sampling (health care centers)
Costa et al [31], United Kingdom	Focus groups, semistructured interviews	Thematic analysis (NVivo v.9); N/I	Background: low socioeconomic status Age=2-3 years Asian and White European parents: n=17, mean age 30.36 SD (6.9) years, 100% (17/17) mothers	Purposive sampling (children’s centers)
Phillips et al [32], United Kingdom	Focus groups, semistructured interviews	Thematic analysis, inductive approach; N/I	Background: highly deprived areas Age=3-4 years Parents: n=11, mean age 29 (SD N/I) years, 100% (11/11) mothers	Purposive sampling (children’s centers, nurseries, preschools)
Ek et al [42], United States	Individual semistructured interviews	Thematic analysis, inductive approach; N/I	Background: urban preschools Age=3-4 years Parents: n=10, mean age 38.9 (SD 5.2) years, 91% (9/10) mothers	Purposive selection of schools (posters)

^a^N/I: not informed.

**Table 2:** Characteristics of included studies (children).

Author, country	Method of data collection	Method of analysis (software); paradigmatic approach	Participants’ details (background, age, parents’ details)	Place and methods of recruitment
Creaser et al [33], United Kingdom	Focus groups, semistructured interviews	Thematic analysis, inductive approach (NVivo, QSR International); N/I^a^	Background: families from different ethnicities Age=5-9 years Parents: n=36, mean age 38 (SD 7.7) years, 67% (24/36) mothers	Purposive sampling (social media)
Coknaz et al [34], Germany	Focus groups, semistructured interviews	Thematic analysis, inductive approach (NVivo v.10); N/I	Background: public primary schools Age=8-13 years Parents: n=N/I, mean age N/I	Purposive sampling (from a clinical trial)
De Vet et al [35], the Netherlands	Focus groups, semistructured interviews	Content analysis (ATLAS.ti v 5.2); N/I	Background: primary schools Age=8-12 years Parents: n=19, mean age 42.3 (SD 4.1) years, 95% (18/19) mothers	Purposive sampling (letter)
Dixon et al [36], New Zealand	Focus groups	Inductive approach; N/I	Background: different ethnicity and socioeconomic groups in urban communities Age=10-14 years Maori parents: n=8, mean age N/I Pacific parents: n=24, mean age N/I Others: n=7, mean age N/I	Purposive sampling (community and church)
Lindqvist et al [37], United States	Focus groups, semistructured interviews	Latent content analysis; N/I	Background: families Age=7-12 years Parents: n=9, mean age 38.7 (SD N/I), 78% (7/9) mothers	Purposive sampling
Rossi et al [38], Italy	Focus groups, semistructured interview	Content analysis (NVivo), community-based participatory action research	Background: mothers Age=0-14 years^b^ Parents: n=5, mean age N/I, 100% (5/5) mothers	Purposive sampling (public health local program)
Sharaievska et al [39], United States	Semistructured group interviews	Open, axial, selective coding techniques, grounded theory	Background: families in rural communities Age=7-13 years Parents: n=N/I, mean age N/I	Purposive sampling
Sobel et al [40], United States	Nonparticipant observations and semistructured interviews	Inductive-deductive approach; N/I	Background: families playing *Pokémon GO* in public locations Age=2-17 years^b^ Parents: n=87, mean age 42 (SD 7.2) years, 70% (61/87) mothers	Purposive sampling (parks, shopping centers, events, online platforms)
Barnett et al [43], Australia	In-depth semistructured telephonic interviews	Thematic analysis (NVivo), descriptive qualitative approach	Background: N/I Age=9-10 years Parents: n=29, mean age N/I	Purposive sampling (from a clinical trial)
Mackintosh et al [44], Australia	Web-based and face-to-face semistructured interviews	Thematic analysis, inductive approach (NVivo v.12); N/I	Background: families Age=7-12 years Parents (web interview): n=25, mean age N/I, 84% (21/25) mothers Parents (face-to-face interviews): n=10, mean age N/I, 100% (10/10) mothers	Purposive sampling (email)

^a^N/I: not informed.

^b^Some studies mixed ages in the sample and did not provide a separate analysis by age.

**Table 3:** Characteristics of included studies (adolescents).

Author, country	Method of data collection	Method of analysis (software); paradigmatic approach	Participants’ details (background, age, parents’ details)	Place and methods of recruitment
Carrion et al [41], Spain	Focus groups	Content analysis, phenomenological approach	Background: parents from public or charter schools Age=13-15 years Parents: n=10, mean age N/I, 50% (5/10) mothers	Purposive sampling (schools)
Lindqvist [45], Sweden	Individual semistructured interview	Latent content analysis (NVivo, QSR International), empowerment	Background: families of a municipality of North Sweden Age=13-15 years Parents: n=10, mean age N/I, 60% (6/10) mothers	Purposive sampling (from an intervention)
McMichael et al [46], United Kingdom	Semistructured interview	Framework analysis, Medical Research Council *(*MRC) framework	Background: families Age=13-17 years Parents: n=18, mean age 53 (SD 3) years, 72% (13/18) mothers	Purposive sampling (social media, schools, university, emails, and posters)

### Study Quality

The assessment of the 18 studies included in this systematic review is presented in Multimedia Appendix 2. Only 1 (6%) study [31] met all 10 items in the JBI-QARI checklist, 8 (44%) studies [20,30,32,33,40-42,46] met 9 items, 8 (44%) [35-39,43-45] met 8 items, and 1 (6%) [34] met 5 items. No studies were rated as “excluded”; thus, none was excluded based on methodological quality. The main weaknesses were a lack of clarity and a lack of reporting on the researcher’s influence on the study and vice versa [20,30,34,35,39,40,43-45]. Other limitations were that participants and their voices were not adequately represented in 3 (17%) studies [34,36,38] and that there was no congruity between the stated philosophical perspectives and the research questions or methodology [34,35].

### Synthesized Findings

We identified 4 main themes (Textbox 1) in terms of parents’ perceptions of PA electronic devices: usefulness, advantages, general perceptions, and acceptability (barriers and facilitators). The main results are shown in Table 4, and proofs are shown in Multimedia Appendix 3.

**Themes and subthemes describing parents’ perceptions of physical activity (PA) electronic devices.**

**Usefulness of PA electronic devices**

- PA promotion and PA in special moments
- Learning of skills and transferability to real life

**Advantages of PA electronic devices**

- Increase in motivation
- Awareness of behaviors
- Family bonding
- Socialization with peers

**General perceptions**

- Electronic devices for health promotion
- Preferences for real-life activities or active screen time
- Concerns: content, addiction, negative emotions, isolation, conflicts, limits

**Acceptability (barriers and facilitators)**

- Lack of time and stress
- Price
- Lack of space at home
- Discomfort/discomfort
- Difficulties with electronic devices or understanding feedback given by the app
- No new activities/suggestions
- Lack of use/interest after novelty
- Attractiveness (high technology, good graphs, good quality, videos)
- Gamification (competition, challenges, goals, and rewards) and fun
- Teacher and school support
- Ease of use
- Durability
- Integrated into daily routines

**Table 4:** Summary of findings.

Participants included, author, country		Area of inquiry/aims	Intervention/exposure	Main results
**Preschoolers**				
	McCloskey et al [20], United States	To explore parents’ beliefs about preschoolers’ use of mobile devices and the acceptability and perceptions of a PA^a^ intervention	Jungle Gym: a mobile app to encourage PA, focused on movement, motor skills (running, jumping, leaping, etc), and interactions with parents/children	Parents supported the use of mobile apps for PA and reported that they were useful in various situations (eg, on bad-weather days). Parents also expressed concerns about the apps.
	Alexandrou et al [30], Sweden	To explore needs and concerns among Somali, Arabic, and Swedish parents regarding a PA app	MINISTOP 1.0 mobile app: a 6-month program to support parents in promoting PA	Parents found the app useful. Insights into their needs and important features were obtained.
	Costa et al [31], United Kingdom	To assess mothers’ opinions about the feasibility and acceptability of using an activity tracker	ActiGraph GT3Xþ, Actiheart (CamNtech Ltd), ActivPAL3 (PAL Technologies Ltd): 3 activity trackers	Children were most comfortable with ActiGraph and least comfortable with Actiheart. Problems with the devices were the possibility of children taking them off, allergic skin reactions, or discomfort.
	Phillips et al [32], United Kingdom	To examine parents’ acceptability and feasibility of measurement tools to assess PA	ActiGraph GT3X+, ActivPAL4 micro, Actical (Philips Respironics Inc): 3 accelerometers	Parents reported that ActivPAL was the least preferred electronic device (children’s opposition to wearing it on their chest, skin irritation). ActiGraph was the most accepted.
	Ek et al [42], United States	To explore parents’ needs and perceptions of a PA app in a school setting	Mobile phone app to promote PA in a school setting	Parents reported the need for interactive features, problem-solving tasks, creativity, and music and dance activities and had a positive attitude toward the app. Children found activities more fun when adults participated.
**Children**				
	Creaser et al [33], United Kingdom	To examine parents’ acceptability of using wearables in a family setting	Fitbit Alta HR for 4 weeks, ActiGraph GT3X+	Fitbit was considered easy and enjoyable to use, but its perceived impact on PA was mixed. Most parents were willing to purchase a wearable.
	Coknaz et al [34], Germany	To analyze the feelings and perspectives of parents about active video games	Nintendo Wii® sports (boxing, tennis, golf, baseball, bowling, skiing, aerobics, running, water skiing, etc) for 50-60 minutes, 3 days/week, 12 weeks	Parents believed that active video games might help in physical changes, socializing, and intellectual and personal development of children.
	De Vet et al [35], the Netherlands	To explore parents’ perceptions and opinions about active video games	Active video games	Parents had a positive attitude toward active and interactive video games. Some parents were less restrictive with them.
	Dixon et al [36], New Zealand	To explore parents’ perceptions of active video games and the probability of sustained engagement	Active video games (eg, EyeToy^TM^, Dance Mat)	Parents supported active video games. They preferred nonviolent and sporty video games. Benefits, such as increased PA, improved fitness, and increased socializing, were reported.
	Lindqvist et al [37], United States	To explore parents’ perceptions of playing *Pokémon GO*	A gamification-inspired program using the *Pokémon GO* mobile game	Parents found that the game promotes PA. They were less likely to limit the time spent on this game. They suggested new features and concerns about safety.
	Rossi et al [38], Italy	To explore parents’ perceptions of a mobile app	Multimodal app for parents’ mobile phones to promote children’s health, including PA	Mothers had a positive attitude toward the app and made suggestions (feedback, geolocalization, and attractive features).
	Sharaievska et al [39], United States	To explore the perception of a PA tracker	PA-tracking electronic device (Fitbit Zip), which each family member was asked to wear for 2 weeks	Parents reported minimal changes in PA because of a lack of interest or an already active lifestyle. The electronic device provided more awareness.
	Sobel et al [40], United States	To explore parents’ perceptions of an app that promotes outdoor PA and to explore how they play with children	*Pokémon GO*	Parents reported an increased level of PA and valued how play led to family bonding. Concerns about safety and limits of gameplay emerged.
	Barnett et al [43], Australia	To identify parents’ perceptions of active video games for development of movement skills	Active video games	Parents were skeptical of the capacity of video games to contribute to skill development and preferred real sports.
	Mackintosh et al [44], Australia	To explore parents’ perceptions of the acceptability and usability of wearable activity trackers to monitor PA	KidFit (X-Doria International) worn by each child for 4 weeks	Parents reported that the activity tracker is easy and useful. Barriers (lack of real-time feedback and difficulties in interpreting information) and suggestions (visual display, self-monitor activity, goal setting, and challenges) were identified.
**Adolescents**				
	Carrion et al [41], Spain	To explore parents’ perceptions, values, and preferences regarding mobile apps to promote PA	PEGASO Fit for Future: a mobile app to promote a healthy lifestyle, including PA, through gamification and family connections	Parents valued mobile apps for health promotion. They preferred apps that promote activity and interactions and include gamification and rewards.
	Lindqvist [45], Sweden	To describe parents’ perceptions of an empowerment-inspired PA intervention via mobile phones	Empowerment-based intervention via Short Messaging Service (SMS)	Parents found that children felt involved in the process and reported that social support and encouragement had an impact on PA. Goals and rewards could be motivating for PA.
	McMichael et al [46], United Kingdom	To understand parents’ views of PA, gaming, and virtual reality in PA interventions	vEngage project active virtual reality	Parents had a negative perception of gaming and preferred real-world PA. They reported the benefits of active games (socializing, motor skills, moving) and concerns (eg, addiction).

^a^PA: physical activity.

#### Parents’ Perceptions of the Usefulness of PA Electronic Devices

The first theme reported was the main usefulness that eHealth technologies might have. The core concepts that support this theme included PA promotion and the learning of skills.

Parents perceived electronic devices as useful for increasing PA levels [34,35,37,39,40,44,45]; for example, parents reported that the *Pokémon GO* mobile game encourages children to be more active and promotes taking long walks through the neighborhood [37,40]. Alternatively, PA is not possible in specific moments when outdoors, for example, on bad-weather days [20]. Regarding activity trackers, parents reported that wearing the electronic device makes the children more motivated to accomplish daily step recommendations or take walks [33,39,44]. However, some parents said that their children, especially younger children, were physically active enough and so did not benefit much from the apps [43].

Regarding motor skills, such as balance or hand-eye coordination, some studies [20,35,40,43] reported that children show improvement and that those skills can be transferable to real sports [43]. In addition, they could learn how to score and follow the rules of some sports [43]. Furthermore, some parents found that eHealth might improve other skills, such as logical thinking and cognitive development [34,35]. In contrast, other parents were skeptical of the transferability of skills learned in video games to a real-life context, and they felt that it is unlikely that their children would benefit from learning skills from virtual apps [43].

#### Parents’ Perceptions of the Advantages of PA Electronic Devices

The advantages of PA electronic devices that parents reported included an increase in motivation for engaging in real-life sports [39,41,43], more awareness, family bonding, and socialization with peers. For example, playing video games motivated children to engage in real-life sports [33,35,37,40,43,44]. Moreover, eHealth apps were useful for parents to become aware of their own levels of PA [39,44], and this, in turn, promoted changes in their attitude toward PA and increased their own PA levels [39]. In addition, parents said that using activity trackers made them aware of other interesting habits of their children, such as sleep or heart rate [30,33,44].

Another advantage of some electronic devices that parents highlighted is that they promote socialization [34,35,37,40,46] and cooperation and competition [37,40,45] with peers and family [20,35,37,39,40,44]. Parents also reported that active video games are suitable for playing with the family and an enjoyable activity to do together, reinforcing their bonds [20,35,37,39,40,44]. Other games promoted social interactions by providing users with something in common to talk about [39,40,44-46] or by enabling them to play interactively with others [35,40,46]; these features were particularly important for adolescents. Thus, parents reported how cooperation and social interaction were important factors in continuing to use the apps, since they found the apps fun and motivating [37,39].

#### Parents’ General Perceptions of PA Electronic Devices

The general perceptions of parents about PA electronic devices were grouped into 3 key concepts: attitudes about electronic devices for health promotion, preference for real-life sports or active electronic devices, and concerns about the use of electronic devices.

Generally, parents were prone to using technology for health and educational purposes [20,42,46]. Furthermore, parents reported the desirability of apps being targeted not only at children but also at parents [30]. They suggested tracking their health lifestyles to be important, such as having an agenda or a reminder and the inclusion of health information [30]. Additionally, parents reported a preference for active and social video games or the active use of screens over passive screen time [35,36,46]. For example, active video games, such as Nintendo Wii, were perceived as a healthier alternative to passive screen time [35]. However, parents distinguished between real-life sports and virtual worlds, showing preferences toward playing outside rather than virtual PA [17,36,40,43,47].

In contrast, they also highlighted several concerns and dangers. Many of the parents were worried about violent content in video games, the appropriateness of content for different ages [35,46], concerns about children playing with strangers, safety [40,46], and physical accidents resulting from walking with the phone in hand [37]. In addition, psychological effects, such as anger, frustration, isolation, or addiction, were also reported [35,37,40,46]. Other common issues highlighted were conflicts when playing video games [37] and difficulties in establishing time limits, which increased with age. In that respect, although parents were more positive toward active video games and active screen use, setting limits and supervising screen use were important issues [20,35,37,40,46].

#### Parents’ Perceptions of the Acceptability of PA Electronic Devices

##### Barriers

Some barriers to using PA electronic devices were found. Commonly, parents reported a lack of time to engage in eHealth activities because of their work or children’s schedules [33,39,45]. Others found difficulties in managing extensive health information and reported feeling stressed by trying to follow all the recommendations [30]. Still, others highlighted the high prices of video games and electronic devices [35,36], and some were annoyed by the noise and space the devices occupy at home while playing [36,42,46].

Regarding the physical characteristics of activity trackers, the main issues raised included unsuitability, discomfort caused by a large size, drawbacks of wearable devices, children trying to remove electronic devices [31,32,44], and difficulties with batteries and syncing [44]. The size of the electronic device was especially important for younger children [31,32]. Other issues were difficulties in using activity trackers or understanding the information provided [33]. Several other factors impacted the use and wearability of activity trackers, including forgetting to wear them, having to remove them for certain sports, the lack of real-time feedback [44], and the lack of interest by parents [33,39]. In this sense, some parents said that activity trackers did not promote any new activity [39]. They also highlighted concerns about the lack of use of the electronic devices once they lost their novelty [33,36] and a lack of long-term wear compliance [44].

##### Facilitators

Parents reported several facilitators of the use of PA electronic devices. For example, they showed a preference for cheaper games that they could afford [35]. Other factors that facilitated engagement were the attractiveness of the game or electronic device, whether it uses high-level technology or appealing graphics [33,46], or the inclusion of videos [30,32,35].

Parents also reported that 2 important facilitators that ensure long-term engagement are gamification and fun [32,33,35,37,42,44]. Teacher support was found to be an important factor in engagement [44,45]. Parents said that goals [31,45] and rewards and new challenges [38,39,43,47] are important features—for example, different levels and new challenges to accomplish [47]. In that sense, many of the parents reported that an important feature is for an app to be fun [39,42,43]. To make apps appealing to children, parents recommended including reinforcement, such as treasure hunts or challenges, which might make the apps motivating. Regarding goal setting, the possibility of establishing goals with others, such as family members, peers, or classmates, was also recommended [31,45]. Furthermore, parents suggested that apps provide interaction with professionals, such as online forums [30,38], and be linked to the school curriculum [44], and teacher support was found to be an important factor in engagement [44,45]. Other ideas were links with sports associations and outdoor activities, such as events, active commuting, and geolocalization [38].

For activity trackers, parents reported some important characteristics that facilitate engagement. Most of them highlighted the importance of comfort [31-33,44], considering that an activity tracker should be worn all the time [32], and ease of use so that the children can understand and handle the device on their own [33,44] with an easy-to-use app [33]. Parents also reported the importance of considering the durability and damage resistance of electronic devices, since younger children might break them [32], and the integration of eHealth with their daily routines [33]. Other suggestions for activity trackers were real-time feedback and a complete dashboard showing information about scores, steps with good graphs, and demonstrations [32]. Features such as competition with others, options for new activities, and high-level technology were perceived as important.

#### Age Group Differences

Of the 18 studies, 5 (28%) [20,30-32,42] analyzed the opinions of the parents of preschoolers’ (<5 years old). Generally, parents were less worried about their children’s PA [30] because they perceived them as spontaneously active and preferred outside PA [20,42]. For preschoolers, most parents tried to limit technology as much as they could [20,42] and used PA apps when real PA was not possible [20,30]. Regarding activity trackers, the problems of wearability due to the size of the devices were highlighted [32].

Furthermore, 10 (55%) studies [33-40,43,44] analyzed schoolchildren between 7 and 12 years old. Parents of children in this age group also showed preferences for real PA [43], although they preferred PA apps over passive screen use [35,36]. Parents were worried about content and addiction and the necessity to set limits on screen time [35-37], and they more frequently reported interactive uses of PA electronic devices with peers and family [35,37]. Regarding activity trackers, parents highlighted the requirement of usefulness for children [44] and the importance of PA electronic devices and activity trackers to be designed specifically for children’s use [33].

In addition, 3 (17%) studies [41,45,46] analyzed samples of parents of adolescents and showed that technology could be an effective strategy to connect with adolescents and help them acquire healthier habits [41]. Regarding this age group, parents were more worried about screen time, the time spent in gaming, and the time spent in sedentary pursuits and preferred technology uses that promote health, education, or socializing [45,46]. They perceived technology as unavoidable and reported difficulties in limiting screen time [46].

## Discussion

### Principal Findings

To the best of our knowledge, this is the first study that systematically reviews qualitative research that explores parents’ perceptions of electronic devices that promote PA in children and adolescents. Overall, parents perceived electronic devices as useful for PA promotion. Moreover, they found other advantages, such as health promotion, awareness of health behaviors, learning of motor and cognitive skills, increased motivation for PA, and promotion of family and social interactions. Parents also valued some of the features of electronic devices, such as being comfortable, easy to use, active, challenging, and fun. However, some barriers and concerns, such as the risk of addiction, safety issues, or difficulties in setting limits, emerged. Preschoolers’ parents found it less necessary to promote PA and preferred that their children spend time in outdoor activities. In contrast, in the case of older children and adolescents, when screen time increased, parents reported more advantages of using active electronic devices that promote PA.

A previous qualitative study that asked parents about their attitudes toward the use of electronic devices and media reported that parents are concerned about the total amount of time that children engage with electronic devices; specifically, they said that engaging with electronic devices prevents children from being physically active [47]. Additionally, other studies have reported positive attitudes of parents toward the use of electronic devices in children, as parents perceive them as a reality in children’s and adolescents’ lives [48], especially for educational and health purposes [49,50]. Similarly, in our study, parents had positive attitudes toward the use of technology for health purposes, such as promoting PA, and they preferred active electronic devices and dance- or sports-based video games rather than traditional sedentary screens [35] because parents perceive active electronic devices as a healthier alternative to passive screen time. Nevertheless, they preferred real PA or outdoor PA over PA on an electronic device [20,35,46]; thus, PA apps do not substitute but complement traditional forms of PA.

Other concerns that parents had, in addition to the high amount of time spent on electronic devices by children and adolescents, were the risk of addiction; the lack of skills; the emergence of negative emotions, such as anger; and violent or sexual content. These concerns are similar to those shown by previous studies, where parents reported being worried about access to inappropriate content, addiction, and negative emotions [9,47,51,52]. In this study, as in previous studies [47,52], parents perceived difficulties in setting limits on the time spent on electronic devices. Their concerns led them to implement different mediation strategies, such as couse, supervision, active mediation, restrictive mediation, and monitoring, depending on positive or negative attitudes toward media [53]. Along this line, parents reported being less restrictive in the case of active electronic devices, rather than passive ones, that promoted social interactions. Regarding social bonds, strong social and family bonds play a large role in controlling the overuse of electronic devices [52]. In this study, parents liked electronic devices that promoted family interactions to play together or that promoted peer interactions, as they believed that games that promote interactions might mitigate the lack of skills and isolation arising from the overuse of electronic devices.

Regarding age, as in a previous study [54], some differences were found, since electronic device usage and social, cultural, and cognitive experiences are vastly different between a 3-year-old child, an older child, and a teenager. In this study, parents of preschool children found no necessity for PA promotion since they perceived that their children were naturally active and used as few electronic devices as possible. In contrast, a study that analyzed general attitudes toward the use of electronic devices and media exposure in young children found that most parents have positive attitudes toward electronic devices, not only for educational purposes but also for entertainment [48]. This difference might be because our study analyzed only PA electronic devices and parents showed a general tendency to overestimate their children’s PA [55], and thus, they perceived a low necessity of electronic devices to increase PA in their children. As children grow older, parents show increasing concerns about the amount of time spent using electronic devices, due to a substantial increase in hours using electronic devices with age [56]. In older children and adolescents, parents report more conflicts and difficulties in limiting electronic device use, consistent with previous studies [18] in which parents of adolescents have reported that setting limits on electronic device use is often confrontational and frequently escalates into arguments and shouting [57]. Therefore, parents implement different mediation practices [58] to regulate the use of electronic devices according to age, as the needs of children and adolescents change with development. Regarding gender differences, only 1 study showed that girls might engage in different challenges and games than boys [46]; congruently, a previous study found limited evidence of children’s gender differences that precluded us from drawing conclusions [54], suggesting that differences in electronic device use and preferences might be considered in further studies.

Finally, parents reported some barriers that need to be considered in further studies, such as lack of time, stress, and high prices of electronic devices. Specifically for activity trackers, comfort, ease of use, difficulties in understanding the apps, or difficulties in understanding the feedback provided were the most common barriers. Conversely, facilitating factors for engagement included the attractiveness of the app, comfort, and children’s self-efficacy in using the electronic device, similar to a previous study of eHealth programs [21]. Some suggestions provided by parents for new PA electronic devices included goal setting and rewards, usability, comfort, real-time feedback, and activities that promote interactions with friends and family, similar to a previous study [8]. In addition, parents had a favorable attitude toward the promotion of technology-based PA strategies in school contexts, and some also considered the involvement of schools and teachers in interventions and connection with the community [42,44,59].

### Strengths

To the best of our knowledge, this is the first systematic review to synthesize findings from qualitative studies examining parents’ perceptions of PA electronic devices. To ensure that the search process was systematic, an exhaustive search was carried out in specialized databases and gray literature by multiple researchers. This search was reported accurately according to the ENTREQ statement [24]. The meta-aggregation approach [29] was used to extract key themes and proofs, which enhanced the reliability of the data. In addition, data were meticulously documented in a matrix, and an assessment of the methodological strength of the analyzed papers was performed.

### Limitations

This review has some limitations that should be acknowledged. First, there was high heterogeneity in the studies regarding the type of electronic device (mobile phones, activity trackers, exergames, virtual reality), data collection methods, location, duration of interventions, sample recruitment strategies, and the age of users. Along this line, studies considering differences between preschoolers, children, and adolescents are needed because these 3 age groups have different lifestyles, interests, and needs. Furthermore, gender differences between boys and girls were considered only in 1 study [46], which might be a source of bias since girls and boys have different levels of PA and different uses and preferences of technology. Second, most participants in the included studies were mothers, which might be due to mothers still parenting more than fathers; however, further studies considering fathers’ opinions are recommended. Finally, some studies did not include an adequate description of the theoretical paradigm and did not provide information about how the researchers’ background was managed.

### Conclusion

This review explored the perceptions of children’s and adolescents’ parents regarding the use of electronic devices for PA enhancement. Parents reported that PA electronic devices could be an effective way to promote PA in children and adolescents and to overcome barriers, such as bad weather, lack of motivation, or the high rate of sedentarism in this population. In addition, parents prefer games and apps that require PA over traditionally passive games and apps. Parents also reported negative attitudes toward the use of technology in terms of addiction, safety problems, and difficulties in establishing limits, which should be considered in future interventions. These insights might provide researchers with more knowledge of how parents manage, promote, and regulate the use their children make of PA eHealth, the acceptability of interventions, and how they use PA eHealth at home. Some important features to consider in the development of new PA apps and technology-based interventions are the developmental stage, ease of use, appropriate feedback, promotion of socialization, and motivating strategies, such as rewards, challenges, and an appealing appearance.

## Appendix

Multimedia Appendix 1 Search strategy.

Multimedia Appendix 2 Methodological quality of included studies.

Multimedia Appendix 3 Findings extracted from included studies, with verbalization of parents’ responses by theme.

## Abbreviations

ENTREQ: Enhancing Transparency in Reporting the Synthesis of Qualitative Research
JBI-QARI: Johanna Briggs Institute Qualitative Assessment and Review Instrument
PA: physical activity

## References

[1] Auxier B, Anderson M, Perrin A, Turner E (2020). Children's engagement with digital devices, screen time. Pew Research Center.

[2] Kabali HK, Irigoyen MM, Nunez-Davis R, Budacki JG, Mohanty SH, Leister KP, Bonner RL (2015). Exposure and use of mobile media devices by young children. Pediatrics.

[3] Smahel D, MacHackova H, Mascheroni G (2020). EU Kids Online 2020: survey results from 19 countries. EU Kids Online.

[4] Radesky JS, Weeks HM, Ball R, Schaller A, Yeo S, Durnez J, Tamayo-Rios M, Epstein M, Kirkorian H, Coyne S, Barr R (2020). Young children's use of smartphones and tablets. Pediatrics.

[5] (2021). EHealth. World Health Organization.

[6] Middelweerd A, Mollee JS, van der Wal CN, Brug J, Te Velde SJ (2014). Apps to promote physical activity among adults: a review and content analysis. Int J Behav Nutr Phys Act.

[7] Fjeldsoe BS, Marshall AL, Miller YD (2009). Behavior change interventions delivered by mobile telephone short-message service. Am J Prev Med.

[8] Carlin A, Murphy MH, Gallagher AM (2015). Current influences and approaches to promote future physical activity in 11-13 year olds: a focus group study. BMC Public Health.

[9] Reid Chassiakos YL, Radesky J, Christakis D, Moreno MA, Cross C, Council on Communications and Media (2016). Children and adolescents and digital media. Pediatrics.

[10] Schoeppe S, Alley S, Van Lippevelde W, Bray NA, Williams SL, Duncan MJ, Vandelanotte C (2016). Efficacy of interventions that use apps to improve diet, physical activity and sedentary behaviour: a systematic review. Int J Behav Nutr Phys Act.

[11] Böhm B, Karwiese SD, Böhm H, Oberhoffer R (2019). Effects of mobile health including wearable activity trackers to increase physical activity outcomes among healthy children and adolescents: systematic review. JMIR Mhealth Uhealth.

[12] Birch LL, Anzman SL (2010). Learning to eat in an obesogenic environment: a developmental systems perspective on childhood obesity. Child Dev Perspect.

[13] Natale R, Messiah S, Asfour L, Uhlhorn S, Delamater A, Arheart L (2014). Role modeling as an early childhood obesity prevention strategy: effect of parents and teachers on preschool children's healthy lifestyle habits. J Dev Behav Pediatr.

[14] Rhodes RE, Blanchard CM, Quinlan A, Naylor P, Warburton DE (2019). Family physical activity planning and child physical activity outcomes: a randomized trial. Am J Prev Med.

[15] Chen J, Weiss S, Heyman MB, Cooper B, Lustig RH (2011). The efficacy of the web-based childhood obesity prevention program in Chinese American adolescents (Web ABC study). J Adolesc Health.

[16] Hammersley ML, Jones RA, Okely AD (2016). Parent-focused childhood and adolescent overweight and obesity ehealth interventions: a systematic review and meta-analysis. J Med Internet Res.

[17] Hoyos Cillero I, Jago R (2010). Systematic review of correlates of screen-viewing among young children. Prev Med.

[18] Sanders W, Parent J, Forehand R, Sullivan AD, Jones DJ (2016). Parental perceptions of technology and technology-focused parenting: associations with youth screen time. J Appl Dev Psychol.

[19] Council on Communications and Media (2016). Media and young minds. Pediatrics.

[20] McCloskey ML, Thompson DA, Chamberlin B, Clark L, Johnson SL, Bellows LL (2018). Mobile device use among rural, low-income families and the feasibility of an app to encourage preschoolers' physical activity: qualitative study. JMIR Pediatr Parent.

[21] Burrows T, Hutchesson M, Chai LK, Rollo M, Skinner G, Collins C (2015). Nutrition interventions for prevention and management of childhood obesity: what do parents want from an eHealth program?. Nutrients.

[22] Young R, Tully M (2022). Autonomy vs. control: associations among parental mediation, perceived parenting styles, and U. S. adolescents’ risky online experiences. Cyberpsychology.

[23] Noyes J, Booth A, Cargo M, Flemming K, Harden A, Harris J, Garside R, Hannes K, Pantoja T, Thomas J, Higgins JPT, Thomas J, Chandler J, Cumpston M, Li T, Page MJ, Welch VA (2022). Chapter 21: qualitative evidence. Cochrane Handbook for Systematic Reviews of Interventions version 6.3 (updated February 2022).

[24] Tong A, Flemming K, McInnes E, Oliver S, Craig J (2012). Enhancing transparency in reporting the synthesis of qualitative research: ENTREQ. BMC Med Res Methodol.

[25] (2020). Children and digital devices. UNICEF.

[26] Booth A, Noyes J, Flemming K, Moore G, Tunçalp Ö, Shakibazadeh E (2019). Formulating questions to explore complex interventions within qualitative evidence synthesis. BMJ Glob Health.

[27] Lockwood C, Munn Z, Porritt K (2015). Qualitative research synthesis: methodological guidance for systematic reviewers utilizing meta-aggregation. Int J Evid Based Healthc.

[28] Pearson A (2004). Balancing the evidence: incorporating the synthesis of qualitative data into systematic reviews. JBI Reports.

[29] Florczak KL (2019). Meta-aggregation: just what is it?. Nurs Sci Q.

[30] Alexandrou C, Müssener U, Thomas K, Henriksson H, Löf M (2021). Adapting a parental support app to promote healthy diet and physical activity behaviors (MINISTOP) for a multi-ethnic setting: a qualitative study on the needs and preferences of parents and nurses within Swedish child health care. Nutrients.

[31] Costa S, Barber SE, Griffiths PL, Cameron N, Clemes SA (2013). Qualitative feasibility of using three accelerometers with 2?3-year-old children and both parents. Res Q Exerc Sport.

[32] Phillips SM, Summerbell C, Hesketh KR, Saxena S, Hillier-Brown FC (2022). Parental views on the acceptability and feasibility of measurement tools used to assess movement behaviour of pre-school children: a qualitative study. Int J Environ Res Public Health.

[33] Creaser AV, Hall J, Costa S, Bingham DD, Clemes SA (2022). Exploring families' acceptance of wearable activity trackers: a mixed-methods study. Int J Environ Res Public Health.

[34] Coknaz D, Mirzeoglu AD, Atasoy HI, Alkoy S, Coknaz H, Goral K (2019). A digital movement in the world of inactive children: favourable outcomes of playing active video games in a pilot randomized trial. Eur J Pediatr.

[35] De Vet E, Simons M, Wesselman M (2014). Dutch children and parents' views on active and non-active video gaming. Health Promot Int.

[36] Dixon R, Maddison R, Ni Mhurchu C, Jull A, Meagher-Lundberg P, Widdowson D (2010). Parents' and children's perceptions of active video games: a focus group study. J Child Health Care.

[37] Lindqvist A, Castelli D, Hallberg J, Rutberg S (2018). The praise and price of Pokémon GO: a qualitative study of children's and parents' experiences. JMIR Serious Games.

[38] Giorgi Rossi P, Ferrari F, Amarri S, Bassi A, Bonvicini L, Dall'Aglio L, Della Giustina C, Fabbri A, Ferrari AM, Ferrari E, Fontana M, Foracchia M, Gallelli T, Ganugi G, Ilari B, Lo Scocco S, Maestri G, Moretti V, Panza C, Pinotti M, Prandini R, Storani S, Street ME, Tamelli M, Trowbridge H, Venturelli F, Volta A, Davoli AM, Childhood Obesity Prevention Working Group (2020). Describing the process and tools adopted to cocreate a smartphone app for obesity prevention in childhood: mixed method study. JMIR Mhealth Uhealth.

[39] Sharaievska I, Battista RA, Zwetsloot J (2019). Use of physical activity monitoring devices by families in rural communities: qualitative approach. JMIR Pediatr Parent.

[40] Sobel K, Bhattacharya A, Hiniker A, Lee J, Kientz J, Yip J (2017). It wasn't really about the Pokémon: parents' perspectives on a location-based mobile game.

[41] Carrion C, Arroyo Moliner L, Castell C, Puigdomènech E, Felipe Gómez S, Domingo L, Espallargues M (2016). Use of the smartphone to promote healthy habits among teen-agers, Spain. Rev Esp Salud Publica.

[42] Ek A, Sandborg J, Delisle Nyström C, Lindqvist A, Rutberg S, Löf M (2019). Physical activity and mobile phone apps in the preschool age: perceptions of teachers and parents. JMIR Mhealth Uhealth.

[43] Barnett LM, Ridgers ND, Hanna L, Salmon J (2013). Parents’ and children’s views on whether active video games are a substitute for the ‘real thing’. Qual Res Sport Exerc Health.

[44] Mackintosh KA, Chappel SE, Salmon J, Timperio A, Ball K, Brown H, Macfarlane S, Ridgers ND (2019). Parental perspectives of a wearable activity tracker for children younger than 13 years: acceptability and usability study. JMIR Mhealth Uhealth.

[45] Lindqvist A (2017). Physiotherapists enabling school children's physical activity using social cognitive theory, empowerment and technology. Eur J Physiother.

[46] McMichael L, Farič N, Newby K, Potts HWW, Hon A, Smith L, Steptoe A, Fisher A (2020). Parents of adolescents perspectives of physical activity, gaming and virtual reality: qualitative study. JMIR Serious Games.

[47] Dorey E, Roberts V, Maddison R, Meagher-Lundberg P, Dixon R, Ni Mhurchu C (2010). Children and television watching: a qualitative study of New Zealand parents' perceptions and views. Child Care Health Dev.

[48] Vittrup B, Snider S, Rose KK, Rippy J (2014). Parental perceptions of the role of media and technology in their young children’s lives. J Early Child Res.

[49] Ihmeideh F, Alkhawaldeh M (2017). Teachers' and parents' perceptions of the role of technology and digital media in developing child culture in the early years. Child Youth Serv Rev.

[50] Lupton D (2021). Young people's use of digital health technologies in the global north: narrative review. J Med Internet Res.

[51] Maxwell J, Kamp J, Cullen T (2021). Parent perceptions of technology use in K-12 classrooms. SRATE Journal.

[52] Buabbas A, Hasan H, Shehab AA (2021). Parents' attitudes toward school students' overuse of smartphones and its detrimental health impacts: qualitative study. JMIR Pediatr Parent.

[53] Nikken P, Schols M (2015). How and why parents guide the media use of young children. J Child Fam Stud.

[54] Goh WL, Bay S, Chen VH (2015). Young school children’s use of digital devices and parental rules. Telematics and Informatics.

[55] Kippe K, Marques A, Martins J, Lagestad PA (2022). Parents' inadequate estimate of their children's objectively physical activity level. Children (Basel).

[56] Shalani B, Azadfallah P, Farahani H (2021). Correlates of screen time in children and adolescents: a systematic review study. J Mod Rehabil.

[57] Hattersley LA, Shrewsbury VA, King LA, Howlett SA, Hardy LL, Baur LA (2009). Adolescent-parent interactions and attitudes around screen time and sugary drink consumption: a qualitative study. Int J Behav Nutr Phys Act.

[58] Livingstone S, Helsper EJ (2008). Parental mediation of children's internet use. J Broadcast Electron Media.

[59] Rioux C, Konkin A, MacKinnon AL, Cameron EE, Tomfohr-Madsen LM, Watts D, Roos LE (2022). Parent preferences for peer connection in eHealth programs. iproc.

