# Supplementary material for: Parents’ Perceptions of Children’s and Adolescents’ Use of Electronic Devices to Promote Physical Activity: Systematic Review of Qualitative Evidence
Source: JMIR Mhealth Uhealth. 2023 Jul 20;11:e44753. doi: 10.2196/44753 (PMC10401398; doi:10.2196/44753)
Supplement: Multimedia Appendix 1 [file mhealth_v11i1e44753_app1.docx]

Multimedia Appendix 1. Search strategy.

| **Database** | **Strategy** |
| --- | --- |
| **PubMed** | (((qualitative[Title/Abstract] OR "qualitative research"[Title/Abstract]OR barriers[Title/Abstract] OR facilitators[Title/Abstract] OR perceptions[Title/Abstract] OR experiences[Title/Abstract]) AND (parents[Title/Abstract] OR father[Title/Abstract] OR mother[Title/Abstract] OR family[Title/Abstract] OR familiar[Title/Abstract] OR relative[Title/Abstract])) AND (sports[Title/Abstract] OR "physical activity"[Title/Abstract] OR exercise[Title/Abstract] OR "physical activity promotion"[Title/Abstract] OR "health promotion"[Title/Abstract])) AND (app[Title/Abstract] OR device[Title/Abstract] OR ehealth[Title/Abstract] OR health[Title/Abstract] OR mobile[Title/Abstract] OR smartphone[Title/Abstract] OR application[Title/Abstract] OR smartwatch[Title/Abstract] OR technology[Title/Abstract] OR computer[Title/Abstract] OR exergames[Title/Abstract] OR "virtual reality"[Title/Abstract] OR gaming[Title/Abstract] OR "videogame"[Title/Abstract] OR internet [Title/Abstract]) |
| **SPORTDiscuss** | "AB ( app OR device OR ehealth OR mhealth OR mobile OR phone OR smartphone OR application OR smartwatch OR technology OR computer OR exergames OR "virtual reality" OR gaming OR "video games" OR videogaming OR exergaming OR "exer gaming" OR "exer game" OR internet ) AND AB ( sports OR "physical activity" OR exercise OR "physical activity promotion" OR "health promotion" ) AND AB ( parents OR father OR mother OR family OR familiar OR relative ) AND AB ( qualitative OR "qualitative research" OR barriers OR facilitators OR perceptions OR experiences ) |
| **Scopus** | ( TITLE-ABS-KEY ( sports OR "physical activity" OR exercise OR "physical activity promotion" OR "health promotion" ) AND TITLE ( app OR device OR ehealth OR mhealth OR mobile OR phone OR smartphone OR application OR smartwatch OR technology OR computer OR exergames OR "virtual reality" OR gaming OR "video games" OR videogaming OR exergaming OR "exergaming" OR "exer game" OR "internet" ) AND TITLE-ABS-KEY ( parents OR father OR mother OR family OR familiar OR relative ) AND TITLE-ABS KEY ( qualitative OR "qualitative research" OR barriers OR facilitators OR perceptions OR experiences ) AND TITLE-ABS-KEY ( children OR adolescent OR youth OR child OR preschoolers ) ) |
| **Web of Science** | ((((TI=(app OR device OR ehealth OR mhealth OR mobile OR phone OR smartphone OR application OR smartwatch OR technology OR computer OR exergames OR "virtual reality" OR gaming OR "video games" OR videogaming OR exergaming OR "exer gaming" OR "exer game" OR internet)) AND AB=(sports OR "physical activity" OR exercise OR "physical activity promotion" OR "health promotion")) AND AB=(parents OR father OR mother OR family OR familiar OR relative)) AND AB=(qualitative OR "qualitative research" OR barriers OR facilitators OR perceptions OR experiences)) AND AB=(children OR adolescent OR youth OR child OR preschoolers) |
| **OpenGrey** | ((((qualitative OR "qualitative research" OR barriers OR facilitators OR perceptions OR experiences) AND (parents OR father OR mother OR family OR familiar OR relative)) AND (sports OR "physical activity" OR exercise OR "physical activity promotion" OR "health promotion")) AND (app OR device OR ehealth OR health OR mobile OR smartphone OR application OR smartwatch OR technology OR computer OR exergames OR "virtual reality" OR gaming OR "video game" OR internet) AND (child OR children OR adolescents OR youth) |
| **DeepBlue** | (app OR device OR ehealth OR mhealth OR mobile OR phone OR smartphone OR application OR smartwatch OR technology OR computer OR exergames OR "virtual reality" OR gaming OR "video games" OR videogaming OR exergaming OR "exergaming" OR "exer game" OR internet) AND (sports OR "physical activity" OR exercise OR "physical activity promotion" OR "health promotion") AND (parents OR father OR mother OR family OR familiar OR relative) AND (qualitative OR "qualitative research" OR barriers OR facilitators OR interview OR perceptions OR experiences) AND (children OR adolescent OR youth OR child OR preschoolers) |
