# Supplementary material for: Parents’ Perceptions of Children’s and Adolescents’ Use of Electronic Devices to Promote Physical Activity: Systematic Review of Qualitative Evidence
Source: JMIR Mhealth Uhealth. 2023 Jul 20;11:e44753. doi: 10.2196/44753 (PMC10401398; doi:10.2196/44753)
Supplement: Multimedia Appendix 2 [file mhealth_v11i1e44753_app2.docx]

Multimedia Appendix 2. Methodological quality of included studies.

| **Reference** | **Q1** | **Q2** | **Q3** | **Q4** | **Q5** | **Q6** | **Q7** | **Q8** | **Q9** | **Q10** |
| --- | --- | --- | --- | --- | --- | --- | --- | --- | --- | --- |
| Alexandrou et al., 2021 | Y | Y | Y | Y | Y | Y | N | Y | Y | Y |
| Barnett et al., 2014 | Y | Y | Y | Y | Y | N | N | Y | Y | Y |
| Carrion et al., 2016 | Y | Y | Y | Y | Y | N | Y | Y | Y | Y |
| Coknaz et al., 2019 | U | Y | Y | Y | N | N | N | N | Y | Y |
| Costa et al., 2013 | Y | Y | Y | Y | Y | Y | Y | Y | Y | Y |
| Creaser et al., 2010 | Y | Y | Y | Y | Y | N | Y | Y | Y | Y |
| De Vet et al., 2014 | Y | Y | Y | Y | Y | N | N | Y | Y | Y |
| Dixon et al., 2010 | Y | Y | Y | Y | Y | N | Y | N | Y | Y |
| Ek et al., 2019 | Y | Y | Y | Y | Y | N | Y | Y | Y | Y |
| Lindqvist 2017 | U | Y | Y | Y | Y | U | N | Y | Y | Y |
| Lindqvist et al., 2018 | Y | Y | U | Y | Y | N | Y | Y | Y | Y |
| Mackintosh et al., 2019 | Y | Y | Y | Y | Y | U | N | Y | Y | Y |
| McCloskey et al., 2018 | Y | Y | Y | Y | Y | Y | N | Y | Y | Y |
| McMichael et al., 2020 | Y | Y | Y | Y | Y | N | Y | Y | Y | Y |
| Phillips et al., 2022 | Y | Y | Y | Y | Y | N | Y | Y | Y | Y |
| Rossi et al., 2020 | Y | U | Y | Y | Y | Y | Y | N | Y | Y |
| Sharaievska et al., 2019 | Y | Y | Y | Y | Y | N | N | Y | Y | Y |
| Sobel et al., 2017 | Y | Y | Y | Y | Y | Y | N | Y | Y | Y |

Y= yes, indicates a clear statement appears in the paper which directly answers the question.

N= no, indicates the question has been directly answered in the negative in the paper.

U= unclear indicates there is on clear statement in the paper that answers the question or there is ambiguous.

Critical appraisal questions for qualitative studies:

Q1. Is there congruity between the stated philosophical perspective and the research question or methodology?

Q2. Is there congruity between the research methodology and the research question or objectives?

Q3. Is there congruity between the research methodology and the methods used to collect data?

Q4. Is there congruity between the research methodology and the representation and analysis of data?

Q5. Is there congruity between the research methodology and the interpretation of results?

Q6. Is there a statement locating the researcher culturally or theoretically?

Q7. Is the influence of the researcher on the research, and vice-versa addressed?

Q8. Are participants, and their voices, adequately represented?

Q9. Is the research ethical according to current criteria or, is there evidence of ethical approval by an appropriate body?

Q10. Do the conclusions draw in the research report flow from the analysis, or interpretation of the data?
