# Supplementary material for: Parents’ Perceptions of Children’s and Adolescents’ Use of Electronic Devices to Promote Physical Activity: Systematic Review of Qualitative Evidence
Source: JMIR Mhealth Uhealth. 2023 Jul 20;11:e44753. doi: 10.2196/44753 (PMC10401398; doi:10.2196/44753)
Supplement: Multimedia Appendix 3 [file mhealth_v11i1e44753_app3.docx]

Multimedia Appendix 3. Findings extracted from the included studies with verbalization of parent’s verbalization by themes.

| **USEFULNESS OF DEVICES** |
| --- |
| **PA physical activity promotion** |
| “He [5-year-old child] has wanted to do more, even though he does enough… he likes to be physical anyways but looking at the Fitbit definitely helped” “Normally we go out on the weekend anyway, but this was another pusher for us to go out and get our 10,000 steps”  “Sometimes if I’d already got 10,000 steps in the morning, I’d go for like 28,000” (Creaser et al., 2022) |
| “We went on a walk last night, all three of us. I think we were outside one hour. Earlier, we have never gone walking that far and especially my son has not earlier been the one pushing us to go out walking.” (Lindqvist et al., 2018) |
| **PA for special moments** |
| “When the weather is bad and the children cannot get outside” (Dixon et al., 2010) |
| “I think that would be good for maybe wicked cold, snowy days or really rainy, funky days. It still gets them up and moving.” (McCloskey et al., 2018) |
| **Learning of skills** |
| “So, as I said, a bit of hand-eye coordination with some of the games, like the bowling. They’ve got to work out what if I put the ball here?” “Good for balance and that sort of thing. Helping with their sport in a way.” “Just coordinating themselves like I know with some of those there’s a stepping one and you have to match the right feet with the steps on the Wii” (Barnett et al., 2014) |
| “Let me tell you how we are playing tennis. My son and I are through the entire living room. She just moves her wrist. And guess who’s winning.”  “And when they really need to think logically about how they can make something work. I like that in a game.” (Devet et al., 2012) |
| “She has gotten better grades since she started to be more physically active. I’m thinking, those who exercise a lot and they are so good at school, too, but it’s surely because they are alert and energetic. It gives energy but it takes time. So, it's good that they are physically active, it really is important.” (Lindqvist et al., 2017) |
| **Transferability to real life** |
| “Having a parent teaching you how to throw and catch a ball is a different experience to… I suppose a computer is not really involved in what you’re doing… fantastic, that’s great, but it’s not a real praise for the child… I think like they don’t get to feel catching a ball, they just have been told that they’ve caught it.” “I don’t think [AVGs can help motor skill development], no. Because I think catching a ball is something you’ve actually physically got to do. I don’t think a game can actually give you that feeling of throwing and catching a ball. You might be doing the actions but to actually do it, real life, is very different” (Barnet et al., 2014) |
| **OTHER ADVANTAGES OF PA DEVICES** |
| **Increase in motivation for PA** |
| “It’s more of an encouragement to get them out there and do the real thing”  “I don’t really like bowling, but they love it because they like playing it on the Wii. She’s learning the skill, and then to score as well.” (Barnett et al., 2014) |
| “My daughter loves to play golf [Wii Sports]. And guess what? She gets stimulated to ask for baseball and golf sets. She asks for all kinds of sports gear.” (Devet et al., 2012) |
| **Gains in awareness of behaviours** |
| “Things that I haven’t considered before like the sleep states were the most interesting” “It was really interesting to see that heart rate breakdown which I found was really good with the Fitbit app” “We were saying to her that you need to sleep more hours coz she’s too busy reading on a night instead of going to sleep and if you check on the app” (Creaser et al., 2022) |
| “I was interested in looking at her sleep habits on the app, rather than so much her steps, because I already knew she was quite active” (Lindqvist et al., 2018) |
| “It made you a little more aware maybe when you thought you got enough exercise but maybe you didn’t quite. I think it did make me more” (Sharaievska et al., 2019) |
| **Family bonding** |
| “It’s not just the kids who are playing. Mom and dad can play along! And they really like it when you do. I like it.” (De Vet., 2012) |
| “In the evening at least, we talked about the steps and what they were doing and what they did for PE and things like that.” “At dinner we would usually look at our fitbits and all say how many steps we did.” (Sharaievska et al., 2019) |
| “We go out for nightly long walks as a family… We stop when we see Pokémon and they help to catch them. They get really excited to go out for walks, so I love taking them.” “Yeah, we were both running together, and she was looking at me, and she was smiling. It was just a great experience” (Sobel et al., 2017) |
| **Socialization with peers** |
| “When they are playing with two, three, four people. It’s just fun. Just like with a board game. They’re communicating, everything is pushed aside.” |
| “I believe that cooperation, when everyone does the same thing, it ties people together. One has something in common to talk about.” (Lindqvist et al., 2017) |
| “My boy has had difficulties interacting with peers. Most of them like to play football. Now he has found friends with the same interests, and his social skills grow every day.” (Lindqvist et al., 2018) |
| “It reinforces that we have common interests and helps us to have a good dialogue as we’re sharing this experience.” “Something in common to talk to the kids [at camp] about, game gives him the opportunity to fit in with kids and talk about it.” “It’s just helping us find a common thing we can do together as a mom and a boy, and that’s really awesome for me. I’m excited about that. I like that he wants to share with me and talk to me about it.” (Sobel et al., 2017) |
| **GENERAL PERCEPTIONS OF PA ELECTRONIC DEVICES** |
| **Health promotion** |
| “When I was a bit stressed… Let’s go for a walk and let’s count how many steps we can get on this” (Creaser et al., 2022) |
| “Technology is here, it’s not going away, and that’s fine if it has benefits for his health, then, yes, I’d always be up for that.” (McMichael et al., 2020) |
| **Preference for “real life” activities** |
| “It can teach you a fundamental basic, but it doesn’t actually prepare you for the real thing … or the basics behind it, but it doesn’t make up for it, definitely not.” (Barnet et al., 2014) |
| “I don’t think active games could replace playing outside and his usual physical activity. Sometimes I see him playing baseball at the Wii while we do have this beautiful bat. That’s just hilarious” (Costa et al., 2013) |
| “I love him engaging in the fantasy world, but I also worry a little bit about starting to see the virtual world as the real world” (Dixon et al., 2010) |
| “You can’t learn how to ride a bike from a phone.” (McCloskey et al., 2018) |
| “I would see it as inferior to physical activity in the real world.” (McMichael et al., 2020) |
| **Preference for active screen time** |
| “We do have a Wii, we purchased it. Why? Because we thought at least you can be active with it” “I like the Wii better, for myself and the kids, as for instance a Nintendo DS. With that you only sit with that thing. ... The advantage of the Wii is that it’s much more active. They don’t sit but are really busy. I like that.” (Devet et al., 2012) |
| “Even if it’s screen time, it’s better than when he’s playing Minecraft.” “I like that. It’s less screen time. They’re not just buried in [the screen] the whole time.” (Sobel et al., 2017) |
| **Concerns: dangers, addiction, negative emotions, isolation, conflicts, limits** |
| “She gets all sort of things that I don’t want her to look at, pornography, a lot of violence” (Dixon et al., 2010) |
| “When I notice that gets annoyed, then we say ‘okay let’s quit the game’. Sometimes, he can’t get passed a level and he gets angry.” “I think that children get a bit isolated.” “There are particular games, in which they get totally lost ... that it becomes the only thing, they say ‘I just want to play a bit more’ and they get totally absorbed.” (Devet et al., 2012) |
| “One hour as well. You play the [Nintendo] DS or the Wii or play half an hour with both. But no longer, it is in the living room, so I keep control. I can see what they play, I can see what they click at.” (Costa et al., 2013) |
| “It is bad that the game is built on having the phone in the hand when moving around since it makes it ok for the children to have the phone in the hand in other situations too, walking around with the phone makes you not pay attention to your surroundings. My son can go downstairs with his eyes on the phone, and he could break his neck falling down” (Lindqvist et al., 2018) |
| “My sister sent a message that a really rare Pokémon was in the city, and my son really wanted to go there, and we couldn´t. It ended in a real fight, and he got really sad, which I can understand because he really wanted that. I have also heard from friends about children becoming hysteric, as it is important to them to catch a Pokémon somewhere. It is like an obsessed behavior.” (Lindqvist et al., 2018) |
| “I do think they’re incredibly addictive and then you get issues with trying to manage the time.” “That she might be playing or getting in contact with people who she doesn’t actually know.” “It seems solitary, another solitary thing that, she’ll be up in her room on her own.” (McMichael et al., 2020) |
| “I don’t want my kids to be the dumb dumbs who fall off a cliff or [get] run over by a car because they [are] too engaged.”  “Just the screen, because it’s still screen time.” “I don’t know how to… stop it. It’s a constant.” “Kids just always wanting to have screen time” (Sobel et al., 2017) |
| **ACCEPTABILITY** |
| **BARRIERS** |
| **Lack of time and stress** |
| “My issue was being sort of throughout the lockdown and home-schooling and the fact that I work from home as well, I don’t have time to–to do much physical activity” “I don’t think I have the scope because I work, I do desk work all day” (Creaser et al., 2022) |
| “A bit of encouragement that it is temporary, that you should not stress too much when things are not going the way you would like them to” (Alexandrou et al., 2021) |
| “She has a lot of stuff going on. She is in a play and has rehearsal 3 times a week and swimming twice a week. Just having to be somewhere. Schedule.” (Sharaievska et al., 2019) |
| **Lack of space at home** |
| “It takes up too much space or gets too loud and noisy, even though they need it daily” (Ek et al., 2019) |
| **Price** |
| “I don’t want to pay fifty or sixty euros for a game. Yes, I think those games are really very expensive” “Something which is very expensive and finished in two weeks and then let it be. That’s a waste” (Devet et al., 2012) |
| **Discomfort** |
| “The sticker one (Actiheart) isn’t [good], because of the tabs. Some children get allergy and then you’re saying you can’t even take it off.” “That would be annoying (placing activPAL3 against child’s thigh) And actually that, for the size of his legs … It would be uncomfortable for him.” (Costa et al., 2013) |
| “I found it quite uncomfortable” (Creaser et al., 2022) |
| “So, it slowly would get tighter and tighter, and especially when she’s out running around, it would get really hot, and kind of sweaty underneath the band, because they don’t breathe either.” “Its too bulky. Too clumpy.” “She was uncomfortable wearing it. It wasn’t even providing her with immediate feedback to sort of provide her with intrinsic motivation... [she was like] wow this is annoying, I don’t like it, so I’m turning it off.” (Mackintosh et al., 2019) |
| “They wouldn’t want something stuck to their body like that. I just don’t think they’d like it at all. Plasters don’t stay on very well, let alone” “Something that’s actually attached to the skin might be kind of irritating for some children” “I think it’s too bulky for her for it to be on the wrist, she’s only dinky, so I think it [Actigraph] would be too big for her” (Phillips et al., 2022) |
| **Difficulties of use** |
| “We couldn’t link our accounts as a family, which sort of ruins that experience as a family because it didn’t—that sort of collective didn’t exist” “You had to press quite ferociously for it to either swap slides or for it to turn back on and it’s quite annoying do that every single time you wanted to see the time or the steps” “I did struggle sometimes putting it on to charge and I don’t know if that was me or I don’t know” “The standout reason [for withdrawing from the study] was it was too much time and energy and effort to actually register and get it started in the first place” (Creaser et al., 2022) |
| “...with the syncing, it did take me a while to understand first of all how to sync.” (Mackintosh et al., 2019) |
| **Difficulties understanding feedback given by the app** |
| “I found that the Fitbit really acknowledged activity easily, which my understanding of moderate to vigorous exercise would really be quite a good raised heart rate, but I found on the Fitbit that it seemed to acknowledge activity just going out for walks” (Creaser et al., 2022) |
| **Lack of use/interest/loss of interest after novelty** |
| “I already saw by the end of week 4 the novelty was wearing off” “I didn’t know whether she [7-year-old child] would have the novelty or if that would wear off” (Creaser et al., 2022) |
| “It might be one of those fad things, they use it all the time for the first month, and then it’ll die off slightly.” (McMichael et al., 2020) |
| “We were already pretty active, so I don’t know. We get outside a fair amount, we go biking and running and stuff throughout the week outside.” (Sharaievska et al., 2019) |
| **No new activities/tips** |
| “I mean they could walk gently and do 10,000 steps; it showed how much he was doing. It didn’t—it didn’t teach me how much he should be doing” (Creaser et al., 2022) |
| “I don’t think we did anything new just more often. We didn’t do anything longer just more daily than a few days a week.” (Sharaievska et al., 2019) |
| **FACILITATORS** |
| **Price** |
| “So, I look for games that are on offer.” (Devet et al., 2012) |
| **Attractiveness (high technology, good graphs, good quality, videos)** |
| “Just because they look cool, they’re kind of fun and if it’s explained to them in a way, they can understand they might think it’s really cool” “Just said he wants superpowers, so he’d have it on all day long” (Phillips et al., 2022) |
| **Gamification and fun** |
| “Well, I would kind of like it to have a little game on it” (Creaser et al., 2022) |
| “We stop when we see Pokémon and they help to catch them. They get really excited to go out for walks, so I love taking them.” (Sobel et al., 2017) |
| “Cooperation and involvement make it fun.” (Lindqvist et al., 2017) |
| “If somebody had 101 [score] and somebody had 102, there would be a flurry of activity, and I would be asked to re-sync, so I think there was that little bit of competitiveness.” (Lindqvist et al., 2018) |
| **Inclusion of challenges, goals, and rewards** |
| “I suppose some kind of score, so you could either beat people or beat your own score” (McCloskey et al., 2018) |
| “They loved it, they loved wearing the device, they loved seeing their goals, they just loved the whole thing.” “The goals need to be more meaningful again. What goals does he want to reach?” “Yeah, like a family goal, and maybe cousins or peers or as a classroom.” (Lindqvist et al., 2018) |
| “You get the buzz when you hit 10,000 steps and I quite like that as a sort of motivation technique” (Creaser et al., 2022) |
| “Gathering things to go on treasure hunts are fun and some kind of positive reinforcement like a point system, also, Pokémon Go has a nice design and fun figures–my five-year-old likes it.” (Ek et al., 2019) |
| “What was good about the app that I had was that one got something when one had gone, for example, 150000 steps; one received a medal like in a program. And then a new challenge was unlocked” (Lindqvist et al., 2017) |
| **Easy to use** |
| “I think the app was really kid friendly” “It was really easy to set up as well and like it just works really well…all I had to do were charge it up once a week and take it off when you had a bath that was it” (Creaser et al., 2022) |
| **Comfort** |
| “I think if it’s on a belt and you can put like a jumper over the top or something, they’ll forget it’s there”. “Oh, absolutely, because it’s like thinner it would be good for bedtimes and they might not feel it as much, but the other one I don’t think that would be very good for bed” “Just for practicality of, just so they’re not stressed as well, if they do want to take it off they can quite easily take it off themselves” (Phillips et al., 2022) |
| **Integrated in daily routines** |
| “What I think would be really useful is for you to be able to put in your routine, so you could set things like don’t be vibrating when you’re in school or when you’re at work, but then make sure you prompt me in between these times cos that’s when I am able to do something and when I need encouragement that sort of thing” (Creaser et al., 2022) |
| “They were doing graphs at school, so it was a quite thing for him.” “Teachers were asking them what they were wearing and so they were telling them that they were doing,” (Mackinntosh et al., 2019) |
| **Inclusion competition, and cooperation** |
| “It would be great to have a competition between the classes, rather than amongst each individual kid, because then they’re helping each other along.” “Grade 5 classes, let’s say you´ve got 3 o 4 just pun them against each other.” “I think that peer kind of tracking peer competition would suit some children really well.” (Lindqvist et al., 2018) |
| “You give the other families the Fitbits and like uh some—a few families the Fitbits and see who gets the most—add all their steps and see how—which people get the most steps” (Creaser et al., 2022) |
| “I think it was cool that we were thinking about it and checking on each other. I think we kind of talked about it and looked at it, maybe twice a day or so.” “He [son] is ultra-competitive with it, and it was kind of neat to celebrate with him and be like how did you get that many? So, I think for me as a parent it was fun to see him have this accomplishment and know that there were many days that he had more than I had.” (Sharaievska et al., 2019) |
| **Video and demonstrations** |
| “Yeah, that’d be good, like a video or something to go on, wouldn’t it? That would be handy… Like just say oh if you go onto this YouTube website or whatever, it shows you how to do it, because all right it’s reading something, but sometimes I need showing… something to show me what to do.” (Creaser et al., 2022) |
| “An app that can inspire through videos of different activities and in the preschool both how you can do it indoors and outdoors and pictures and maybe also that these videos are with other children so that the children here can relate to themselves and to the preschool setting.” (Ek et al., 2019) |
| **Age adapted and multiage** |
| “If there’s a kids range focused on kids that would encourage them to wear it more often as well and be a bit more interested in it as well if it was more focused on you know kids range of straps” (Creaser et al., 2022) |
| “That they both can play the game because I also have a younger son. So, I look at the age rating, that they both can play the game together and not individually for the eldest.” (Devet et al., 2012) |
| **Better feedback** |
| “Good to have an app that keeps track of your lifestyle habits, such as sleep, have I achieved my goals? Or ‘Is this how it’s supposed to be?’ Then, it will also be on my agenda, like a reminder to me.” (Alexandrou et al., 2021) |
| “I don’t know if you could have more of a number scale or something like that, you know 1 to 10... 10 is vigorous you know, 5 is moderate, that type of thing” “Explain what active minutes means... is it the same as intensity minutes” (Creaser et al., 2022) |
| **Durability** |
| “Childproof, like hard, like not feeble, if you know what I mean-so they’ll easily break.” “Absolutely, no small parts either we have to say these days. Childproof” (Phillips et al., 2022) |
